# Parenteral iron—Does it increase infection risk?

**DOI:** 10.1111/vox.70241

**Published:** 2026-03-25

**Authors:** Joyisa Deb, Aswin K. Mohan, Suhasini Sil, Suvro Sankha Datta, Yoghini Nagandran, Manideepa Maji, Mohammad Kamrul Hassan Majumder, Saikat Mandal

**Affiliations:** ^1^ Department of Transfusion Medicine & Blood Centre All India Institute of Medical Sciences Guwahati Assam India; ^2^ Department of Transfusion Medicine All India Institute of Medical Sciences Bibinagar Telengana India; ^3^ Department of Transfusion Medicine, Blood Centre Main Hospital All India Institute of Medical Sciences New Delhi India; ^4^ Department of Transfusion Medicine Tata Medical Center Kolkata India; ^5^ Department of Frailty and Ageing Hull University Teaching Hospitals NHS Trust Hull UK; ^6^ Centre for Biomedicine Hull York Medical School, University of Hull Hull UK; ^7^ Department of Haematology Hull University Teaching Hospitals NHS Foundation Trust Hull UK; ^8^ Department of Haematology Portsmouth Hospitals University NHS Trust Portsmouth UK; ^9^ Translational Medical Sciences School of Medicine, University of Nottingham Nottingham UK; ^10^ NIHR Nottingham Biomedical Research Centre Nottingham University Hospitals NHS Trust and the University of Nottingham Nottingham UK; ^11^ University of Leeds Leeds UK

**Keywords:** infection risk, intravenous iron, iron deficiency, sepsis

## Abstract

**Background and Objectives:**

Iron deficiency (ID) and iron deficiency anaemia (IDA) are prevalent conditions impacting various patient populations, both surgical and non‐surgical conditions. The advent of patient blood management (PBM) has promoted intravenous (IV) iron therapy as an alternative to oral iron and blood transfusions. However, concerns remain regarding IV iron's potential association with infection risk. This narrative review critically examines the relationship between parenteral iron therapy and infection risk across various clinical settings. It evaluates various IV iron formulations, their benefits, safety profiles and potential adverse effects, particularly infection‐related complications.

**Materials and Methods:**

A structured literature search was conducted across PubMed, EMBASE, Medline and CINAHL (2014–2024) using pre‐defined keywords. Observational studies and clinical trials relevant to IV iron formulations and infection risk were analysed.

**Results:**

IV iron therapy effectively improves haemoglobin levels and reduces transfusion dependence. Studies in cardiovascular, renal, antenatal and surgical populations suggest that it is difficult to conclude that IV iron therapy significantly increases the risk of infection. Older formulations, high‐dose IV iron therapy and various underlying conditions may elevate infection susceptibility due to increased levels of non–transferrin‐bound iron. Emerging formulations, such as ferric carboxymaltose and ferric derisomaltose, appear to have a more favourable safety profile.

**Conclusion:**

While IV iron remains a cornerstone in ID management, patient‐specific risk factors must be considered. Further research is needed to clarify infection risk variations among different IV iron formulations and patient populations. Optimizing IV iron therapy through individualized approaches may enhance its clinical benefits while minimizing potential adverse effects.

**Highlights**

- Excessive iron levels may increase susceptibility to infections as per literature review, but the evidence remains inconclusive due to confounding factors.
- Emerging strategies to reduce infection risk include using intravenous (IV) iron formulations with lower labile iron content and exploring the role of lactoferrin, iron chelators, nanoparticles and hepcidin mimetics as potential therapeutic options.
- Well‐powered multicentric controlled clinical trials in various clinical populations are needed to define the safest approaches.

## INTRODUCTION

Iron deficiency (ID) is a widespread condition with diverse aetiologies across different age groups and patient demographics. Iron‐deficient patients may experience symptoms regardless of whether anaemia is present. Nutritional ID is a leading cause, particularly affecting women, adolescent populations in lower socioeconomic countries and patient cohorts with gastroenterological ailments [1, 2]. In surgical settings, ID and iron deficiency anaemia (IDA) are associated with poor perioperative and post‐operative outcomes [3]. Traditionally, IDA has been managed using oral iron supplementation and blood transfusion. However, the advent of patient blood management (PBM) programmes in the late 1990s and early 2000s has promoted alternative therapeutic approaches, including intravenous (IV) iron therapy [4].

The U.S. Food and Drug Administration (FDA) has approved several IV iron formulations for specific indications (Table S1) [5]. While oral iron remains the first‐line treatment, IV iron has gained prominence due to its advantages over oral therapy. These include greater increases in haemoglobin levels, serum ferritin and transferrin saturation (TSAT), as well as improved safety and efficacy profiles [6]. IV iron is particularly beneficial in patients where oral iron is contraindicated, such as those with underlying inflammatory conditions, impaired gastrointestinal absorption, or a need for rapid replenishment of iron stores [7]. Newer formulations further improve safety profile and reduce frequency of hospital visits [8].

Iron is an essential element in various biological processes. Including haemoglobin synthesis, myoglobin formation, energy metabolism, neurotransmitter production, collagen synthesis and immune function [1, 9]. Like a double‐edged sword, both ID and iron excess may precipitate infection, highlighting the need for tight homeostatic regulation of iron metabolism [10, 11]. In healthy individuals, iron levels are tightly regulated through multiple mechanisms, with hepcidin playing a central role. Hepcidin creates ‘nutritional immunity’ by limiting free iron availability to both intracellular and extracellular pathogens. Inflammatory responses trigger interleukin(IL)‐6 production, which, in turn, increases hepcidin production. Hepcidin downregulates ferroportin expression, thereby reducing gastrointestinal iron absorption and promoting retention within macrophages. Additionally, unsaturated transferrin binds free iron in the bloodstream and transports it to cells for storage. This coordinated process creates an iron‐deprived environment, restricting iron availability to extracellular pathogens like *Staphylococcus aureus* [12]. Yet, the protective nature of nutritional immunity presents a paradox, for it can inadvertently enhance the virulence of intracellular pathogens, such as *Chlamydia*, *Legionella*, *Salmonella* (*S. typhimurium*) and *Mycobacteria* spp. [13, 14, 15]. Such pathogen class‐specific mechanisms are particularly relevant in the context of co‐infections. For instance, haeme catabolism and the resulting intracellular iron levels confers host protection against the blood stages of *Plasmodium* infection while simultaneously increasing susceptibility to *Salmonella* [16]. Understanding this complex host–microbe relationship is crucial for optimizing IV iron therapy and mitigating infection risks. Whether these principles extend to co‐infections involving other pathogens, while administering parenteral iron remains a critical area of investigation.

IV iron therapy increases the levels of circulating non‐transferrin‐bound iron [1]. Deicher et al. suggested that frequent and high doses of IV iron sucrose adversely affected the intracellular killing capacity of neutrophils and resulted in higher hospitalization rates and lower survival rates of dialysis patients. A subset of the study population with high ferritin levels (>600 μg/L) was found to be less susceptible to iron‐mediated oxidative stress [17]. As IV iron use grows, there is an unmet need to investigate its potential association with infection risk and associated morbidity and mortality. To date, much of the available evidence on this topic has been conflicting and inconclusive. Study by Shah et al. suggests that newer IV iron preparations pose a lower infection risk due to reduced free iron concentrations [18]. There were variations in infection risk on subgroup analysis among different patient groups and based on the iron formulation they received [18]. There are recently conducted observational studies, clinical trials and systematic reviews in different patient groups like kidney transplantation, femur fracture surgery, haemodialysis patients mention that there was no infection risk associated with IV iron infusion [19, 20, 21].

This consensus document examined the published literature critically to determine the potential association between parenteral iron and infection risk in both surgical and non‐surgical patient profiles, particularly in light of the most recent IV iron formulations. This review summarizes the various aspects of IV iron formulations in practice, including the benefits, efficacy and side effects of each formulation and their pathophysiological mechanisms. Finally, all the above information is being used to discuss the risk of infection associated with different IV iron formulations across a range of clinical settings, highlighting gaps in the existing medical literature and proposing areas where further research is needed.

## METHODOLOGY

We performed a structured literature search of the following databases, PubMed (e‐publications ahead of print only), EMBASE, Medline and CINAHL from January 2014 to January 2025, combining relevant keywords that support the research question. The search was repeated by two different individuals on two different dates to limit the risk of missing any relevant piece of literature. The following key words were selected: ‘Intravenous iron’ OR ‘parenteral iron’ OR ‘IV iron’ OR ‘iron infusion’ OR ‘ferric carboxymaltose’ OR ‘Iron sucrose’ OR ‘Iron dextran’ OR ‘Ferric derisomaltose’ OR ‘Low molecular weight iron dextran’ OR ‘Ferumoxytol’ OR ‘Ferric Gluconate’ OR ‘Iron Isomaltoside’ OR ‘Nano particle‐based iron therapy’ AND ‘Infection’ OR ‘Sepsis’ OR ‘Septic shock’ OR ‘Bacteraemia’ OR ‘pneumonia’ OR ‘bronchitis’ OR ‘Cellulitis’ OR ‘Abscess’ OR ‘Pyelonephritis’. Search results were screened to remove duplicates; then the remaining ‘x’ publications were reviewed based on the abstracts published in English language and clearly related to the research question. Full‐text articles were obtained for all the publications and selected if they included original clinical data or clinical experience on IV iron formulation related to infections/sepsis. Our search was restricted to human studies.

We included observational studies or clinical trials conducted on different surgical and non‐surgical conditions irrespective of the type of IV iron formulations used. Additionally, and where appropriate, we integrated different aspects of the working group's clinical experience in this field. Publications without original data on clinical evidence (e.g., reviews, editorials and guidelines) were excluded. Congress abstracts were excluded if the data were subsequently available in a full publication. However, we retained case studies that have reported interesting but rare case findings.

We have focussed on understanding the role of IV iron as a therapeutic agent in surgical and non‐surgical patient profiles. Outcomes considered were development of adverse events like infection, sepsis or related complications in different clinical settings (both surgical and non‐surgical) within 6 months of receiving IV iron formulations. Different available IV iron formula were considered with benefits, efficacy and side effects of each formulation along with their associated pathophysiological mechanisms. Finally, all the above information was used to discuss the risk of infection associated with IV iron in different settings and address the identified areas of unmet need. The significance of use of IV iron in PBM from the perspective of increased infection risk was evaluated.

## DISCUSSION

### Different formulations of IV iron therapeutic agents: Analogous yet clinically distinct

Since the introduction of ferric hydroxide as the first parenteral iron compound at the dawn of the 20th century, IV iron therapy has undergone significant advancements. In 1947, Nissim introduced the concept of encasing iron salts in a carbohydrate shell to reduce toxicity, which enhanced cost‐effectiveness, ease of administration and patient compliance, making IV iron therapy a cornerstone in the management of IDA [22, 23]. The next generation of parenteral iron formulations included the iron core embedded in a dextran polymer, sucrose or gluconate. These compounds are no longer routinely used and are largely replaced by newer IV iron preparations in the early 21st century, including ferumoxytol (approved in the United States), iron isomaltoside (approved in Europe) and ferric carboxymaltose (approved in Europe). These newer formulations offer improved safety profiles and allow for faster administration of larger doses (Table S2) [23, 24, 25, 26, 27, 28, 29, 30, 31, 32, 33, 34].

### Iron therapy in different clinical settings and risk of infection

#### Medical conditions

##### Cardiovascular disease

IV iron therapy is essential for management of ID in cardiovascular diseases, including heart failure (HF) and severe aortic stenosis, where oral iron is often inadequate [35, 36]. While IV iron improves symptoms and functional capacity, concerns exist about increased oxidative stress and infection risk [37].

Kalra et al reported that IV ferric derisomaltose reduced recurrent hospitalizations and cardiovascular death in HF patients with ID [36]. A sub‐analysis revealed a 24% reduction in cardiovascular events and a 21% decrease in all‐cause hospitalizations, without increasing infection risk [38]. Similarly, Jankowska et al suggested that IV iron is beneficial for HF patients without elevating infection risk [37]. Kvaslerud et al is investigating IV iron's effect on physical capacity before trans catheter aortic valve implantation (TAVI) for severe aortic stenosis. A retrospective study on patients with stable chronic HF suggested an improvement in iron profile and quality of life on receiving IV iron therapy [38]. These studies highlight ID as a treatable comorbidity in cardiovascular conditions, with IV iron improving symptoms and exercise capacity, but also reducing cardiovascular events, without increasing the risk of infection [35].

##### Renal diseases

IV iron is widely used to manage anaemia in haemodialysis patients, when oral iron fails. Although it improves haemoglobin levels and reduces erythropoiesis‐stimulating agent (ESA) dependence, infection risk remains debated [39].

Studies by Winkelmayer et al. and Brookhart et al. suggested an association between ferric gluconate and increased infection rates, although confounding factors were present [39, 40]. A randomized controlled trial (RCT) by Bielesz et al. observed higher infection rates with iron sucrose than ferric carboxymaltose, potentially due to frequent administration regimen [41]. Agarwal et al also suggested increased lung and skin infections with IV iron sucrose compared to oral iron in patients with chronic kidney disease, though it was terminated early [42]. In haemodialysis patients, high IV iron doses were linked to more adverse events, including infections [43]. Conversely PIVOTAL (Proactive IV Iron Therapy in Haemodialysis Patients) trial and secondary analysis by Macdougall et al. found no significant association between IV iron and infection risk [44]. Yen et al.'s nationwide cohort study and Ishida et al.'s analysis of the US Renal Data System found no significant link between IV iron and infection risk [45, 46]. Study by Yu et al. mentions all‐cause mortality or adverse events due to infection were not higher among parenteral iron replacement recipients than with oral or no iron therapy recipient Korean haemodialysis patients in a prospective observational study [19]. Another single‐centre retrospective observational study by January et al. reported IV iron did not increase the risk of bacterial infections in the immediate post‐kidney transplant setting [20].

IV iron is a crucial therapeutic for management of anaemia in kidney disorders. Careful monitoring and further research are needed to determine the optimal dosing, timing and specific formulations to use, as well as to understand the long‐term safety profile.

##### Pregnancy

Anaemia in pregnancy is associated with complications for both mother and newborn [47]. While oral iron is preferred, IV iron offers rapid rise in haemoglobin, improved vitality and social functioning [48]. The American Society of Hematology guidelines recommend IV iron in the second half of pregnancy [49]. Iron sucrose and sodium ferric gluconate are listed in FDA category B classification, while low‐molecular‐weight (LMW) iron dextrans is a category C designation [50].

A Malawian RCT found no significant reduction in anaemia prevalence at 36 weeks of gestation or birth weight differences between IV ferric carboxymaltose and oral ferrous sulphate, with almost similar infection rates including malaria (52% vs. 50%) in IV iron therapy arm [51]. The IVON trial (Intravenous versus oral iron for anaemia among pregnant women in Nigeria), conducted in Nigeria, showed IV ferric carboxymaltose was more effective than oral iron in reducing ID, increasing haemoglobin levels, without significant adverse effects or reduction in transfusion needs [52]. A multicentre trial in India found no significant difference in the primary maternal composite outcome (postpartum haemorrhage, puerperal sepsis, prolonged hospitalization). There was a 2% versus 3% need for blood transfusion in iron sucrose and oral iron groups, respectively [53]. Hemminki et al reported more maternal deaths during pregnancy and in the postpartum period, although causality remains unclear [54]. Concurrent inflammation (elevated C‐reactive protein) can be a significant factor in pregnancy. Pasricha et al. reported similar infection rates (7.9% vs. 8.4%) between IV iron and standard care but higher prevalence of inflammation (75% vs. 67.3%) in the IV iron group [55]. While IV iron benefits both mother and neonate, yet before using it on a regular basis, further study is needed to understand the role of underlying inflammation and the pregnancy state in patient outcomes.

##### Other medical settings

Limited number of studies conducted in the paediatric population indicate IV iron therapy (iron sucrose, ferric carboxymaltose) significantly increases haemoglobin levels, with a good safety profile, in infants and young children. Reported adverse events include pyrexia and upper respiratory tract infections; however, no clear predominance of infections was established [56, 57, 58]. A novel nano iron supplement Iron Hydroxide Adipate Tartrate was found non‐inferior to ferrous sulphate in treating paediatric IDA without added infection risk [59].

#### Surgical settings

##### Orthopaedic surgery

A randomized single‐centre trial by Bielza et al. in older hip fracture patients found no added infection risk with iron sucrose compared to placebo [60]. Similarly, an observational study by Hansen et al. reported no increase in post‐operative infections with ferric derisomaltose IV therapy in geriatric hip fracture patients [61]. A multicentre cohort study by Lucas et al. found fewer nosocomial infections in the ferric carboxymaltose group compared to blood transfusion, though statistically insignificant [62]. Another RCT on older patients undergoing hip fracture surgery also showed no increased infection risk with IV iron carboxymaltose [63]. Lasocki et al. reported that derisomaltose and tranexamic acid, if given together for hip fracture can lower transfusion requirement. Infections were the most common adverse effects, occurring equally across study arms [64]. While there are no strong evidence linking IV iron therapy to increased infection risk in orthopaedic patients (including the high‐risk geriatric patients), it has been shown to reduce allogenic transfusion needs and improve haemoglobin levels, which can positively contribute to patient prognosis.

##### Cardiothoracic surgery setting

A single‐blinded RCT by Xu et al. found no increase in infection rates or poor wound healing in post‐operative cardiac valvular surgery patients with functional IDA receiving IV Venofer [65]. Similar trials studying the role of ferric carboxymaltose in cardiac surgery patients showed no infection risk associated with this therapy [66, 67, 68]. An RCT by Friedman et al. in cardiac patients without anaemia found that pre‐operative ferric carboxymaltose, significantly increased post‐operative haemoglobin and reduced transfusion needs [68]. However, it remains debatable if prophylactic ferric carboxymaltose infusion can reduce transfusion requirements, for that depends on key variables such as dosage and timing of administration [66, 68].

##### Other surgical settings

IV iron therapy is widely studied in colorectal cancer, major abdominal, urological and gynaecological oncology surgeries. A single‐centre study by Laso‐Morales et al. in elective colorectal cancer surgery found lower infection rates with ferric carboxymaltose than iron sucrose, possibly due to reduced free iron availability [69]. A similar multicentre RCT found that IV iron significantly increased the haemoglobin levels, though the occurrence of adverse events was not discussed [70]. Studies by Richards et al. on IV ferric carboxymaltose before major open abdominal surgery found no significant infection risk or mortality, whereas Khalafallah et al. reported lower post‐operative infection rates with ferric carboxymaltose [71, 72]. Assouline et al. found no increase in infection risk, inflammatory marker levels or transfusion requirement in liver surgery patients between IV iron and the control group [73]. A retrospective review by Goh et al. found that iron isomaltoside is beneficial for patients with anaemia undergoing urological surgery, including the subcohort of patients with anaemia due to sepsis, with a favourable safety profile [74].

In light of variations in study designs, inherent study qualities or the patient conditions that can precipitate infection or inflammation, it is necessary that the discerning individual approach their interpretation with the utmost prudence and judicious consideration. IV iron administration, both pre‐ and post‐operatively, can improve haemoglobin levels and iron stores in various surgical patient populations, with some studies suggesting a potential to reduce the need for blood transfusions, need for antibiotics, hospitalization duration and improve wound healing.

### Iron therapy and risk of infection

A good iron store is crucial for immune function, yet an excess supply might compromise host immunity. This association stems from observations in conditions like haemochromatosis where high TSAT enhances bacterial proliferation [75]. Recent advancements in iron physiology and newer IV iron formulations have prompted numerous studies evaluating infection risks. Current evidence from both surgical (e.g., cardiopulmonary, urology orthopaedic) and non‐surgical populations does not conclusively demonstrate that IV iron therapy significantly increases the risk of infection or mortality compared to controls [60, 61, 62, 63, 64, 65, 66, 67, 68, 69]. While some meta‐analyses and reviews suggest a possible association between IV iron therapy and increased infection risk, the evidence remains inconclusive and inconsistent, particularly when different clinical settings are considered [18, 76]. Notably, many studies did not designate infection as a pre‐defined outcome variable, hence inducing unmeasured bias. In murine models, IV iron worsened sepsis outcomes [77, 78], but human studies have not established a direct link between high‐dose IV iron and increased mortality; instead, they reflect on underlying comorbidities [79, 80, 81].

Risk factors for infection with iron therapy include advanced age (>60 years), chronic kidney disease, dialysis, prior infections, catheter use, prolonged/high‐dose iron, elevated serum ferritin (>500–1000 ng/mL) and concurrent erythropoietin use [18, 44, 82, 83, 84, 85]. Geographic considerations (e.g., malaria‐endemic regions) and underlying conditions such as inflammatory bowel disease may further influence outcomes [86].

The risk of infection associated with IV iron is multifactorial; however, that should not deter us from using it when indicated. Based on the literature review, we recommend that parenteral iron be used with caution in patients with suspected infection or when multiple risk factors are present, such as advanced age, chronic kidney disease, a history of bacterial infections, catheterization, prolonged iron therapy, concurrent erythropoietin use, high‐dose iron administration, elevated serum ferritin levels or underlying inflammatory bowel disease.

### Non–transferrin‐bound iron: Link between iron therapy and infection risk

Non–transferrin‐bound iron (NTBI) can potentiate growth of pathogens such as *S. typhimurium*, *Escherichia coli* and *Yersinia pestis*, if TSAT is elevated. Animal model data support this mechanism, but human clinical studies remain limited [16, 17, 18]. Therefore, it is critical to evaluate whether surrogate markers of free iron—such as TSAT and serum ferritin are included in clinical studies assessing infection risk with IV iron therapy.

Several trials incorporated these iron markers (e.g., TSAT, serum ferritin) to stratify iron status and guide therapy. The IRONMAN trial used TSAT <20% and a ferritin <100 μg/L to define ID [36]. The PIVOTAL trial allocated patients to high‐ or low‐dose iron groups based on both serum ferritin and TSAT values, with regular monitoring to avoid iron overload [44]. The HiFIT and FORGE trials have used serum ferritin and TSAT, though thresholds varied [64, 87]. The MAINTAIN trial withheld iron therapy when ferritin >700 ng/mL or TSAT >40%, thereby incorporating dynamic monitoring into treatment algorithms [43]. Goh et al. specifically excluded patients with serum ferritin >800 ng/mL, conditions like iron overload, haemochromatosis and inflammatory bowel disease in his study [74]. Some studies included additional markers such as total iron‐binding capacity (TIBC) and hepcidin [42, 88]. However, several studies in this review did not report iron indices as part of their inclusion criteria, monitoring protocol or outcomes [45, 46, 54, 60, 61]. This inconsistency may limit the interpretability of infection risk across studies.

In one study by Umanath et al., TSAT correlated more reliably with iron availability than serum ferritin, particularly in the inflammatory milieu of chronic kidney disease, where ferritin may be elevated, being an acute‐phase reactant. These parameters can be used as valuable tools for infection risk stratification in iron therapy [89].

The ongoing INFER trial, evaluating IV iron polymaltose in haemodialysis patients with elevated ferritin, is expected to provide insight into outcomes at higher iron thresholds [90]. Future studies would benefit from standardized biomarker thresholds and, where feasible, incorporation of direct NTBI measurements to better predict infection risk.

### Context‐specific approach

There is a lack of conclusive evidence confirming the effects of IV iron therapy during active infection or sepsis in human beings. While robust data are lacking to justify withholding of IV iron in such settings, clinical caution is generally advised—particularly in patients with systemic infections or severe inflammatory states who concurrently require iron therapy. The Kidney Disease: Improving Global Outcomes (KDIGO) recommendations emphasize the need for subgroup‐specific vigilance; yet it is important to note that this recommendation is weak and not formally graded [83, 86, 91]. In a retrospective observational study, Ishida et al. evaluated outcomes associated with IV iron therapy during active infection in haemodialysis patients. Recipient of ferric gluconate, was associated with a higher odd of infection‐related readmission, compared to iron sucrose [46]. These hypothesis‐generating findings, underscore the need for well‐designed, prospective studies to more definitively assess the safety and efficacy of IV iron during active infection.

Tight homeostatic regulation of iron metabolism is essential to balance the risks of severe ID and bloodstream infections [12]. Persistent functional ID, disordered iron metabolism and elevated serum TSAT/iron concentrations are negative prognostic factors in Intensive Care Unit (ICU) patients [11, 92]. Supported by literature, IV iron reduces anaemia, red blood cell transfusions and hospitalization rate in specific patient populations [25].

A two‐step approach that involves initial screening with standard iron assays to exclude patients at risk of iron overload, followed by targeted IV iron therapy—may improve safety. Newer IV formulations with low labile iron content and improved safety profiles do not report an increased risk of infection. To minimize infection risk, the lowest effective dose should be administered for ID. Formulations that release lower free iron, such as ferric carboxymaltose and ferumoxytol, are preferable. Evaluating and addressing underlying infections prior to iron therapy can help mitigate the risks [25].

A nuanced, context‐specific assessment of infection risk is essential for different patient groups. It should be identified if IV iron therapy predisposes patients to specific infections, considering microbial iron acquisition mechanisms. A head‐to‐head comparison of different IV formulations, standardized infection definitions, as well as identification of causative micro‐organisms could refine therapeutic strategies. This can be achieved through well‐conducted and adequately powered RCTs. Various confounding factors should be well accounted for, as they may limit result validity.

### Need for future research

1. *Biomarker for mitigating infection risks associated with iron therapy*: Growing evidence highlights the role of iron in infection susceptibility and resistance. Bacterial siderophores derive iron for their sustainability from transferrin, ferritin or lactoferrin [76]. Lactoferrin, an iron‐binding glycoprotein, has demonstrated potential in reducing nosocomial infections in preterm infants, though results in adult ICU settings remain inconclusive [93, 94]. Lactoferrin limits iron availability to micro‐organisms and also regulates iron homeostasis, preventing anaemia and inflammation in pregnancy by multiple mechanisms [95]. Ferritin affects the production of cytokines like IL‐6, IL1β, nitric oxide and other immune cell mediators [96]. Hepcidin/hepcidin‐mimetics reduce bacterial proliferation in animal models by decreasing serum iron levels [97]. Iron chelators reduce parasitic burden in malaria patients and bacterial burden in tuberculosis patients [98, 99]. Additionally, iron nanoparticles in recent times seem to be a promising compound for faster, effective and tissue‐specific targeted delivery of iron in IDA [100]. They possess bactericidal properties by the generation of reactive oxygen species, membrane disruption and cell enzymatic inhibition in micro‐organisms [101]. In summary, patient‐specific approach, biomarker‐driven strategies, single‐dose therapies and safer iron formulations are optimal for mitigating infection risks associated with iron therapy.
2. *Developing multicentric clinical trials*: To strengthen current understanding, robust evidence from well‐designed multicentre clinical trials is needed, incorporating pre‐defined and validated definitions of infection across diverse patient populations. Additionally, systematic reviews and meta‐analyses should be conducted to generate high‐quality evidence across patient groups with varying clinical diagnoses, given the multifactorial nature of infection development. Such evidence would help guide the safe and effective use of this therapeutic intervention, particularly in identifying patients at high risk of infection. These efforts would support informed clinical decision‐making and ultimately improve patient outcomes.

## CONFLICT OF INTEREST STATEMENT

The authors declare no conflicts of interest.

## Supporting information


**Table S1.** FDA approved indications for intravenous (IV) iron therapy [5].
**Table S2.** Different parenteral iron formulations and their salient features.

## Data Availability

Data sharing not applicable to this article as no datasets were generated or analysed during the current study.
