# Supplementary material for: Parenteral iron—Does it increase infection risk?
Source: Vox Sang. 2026 Mar 25;121(7):914–22. doi: 10.1111/vox.70241 (PMC13357008; doi:10.1111/vox.70241)
Supplement: Supplementary file 1 — Table S1. FDA approved indications for intravenous (IV) iron therapy [5]. Table S2. Different parenteral iron formulations and their salient features. [file VOX-121-914-s001.docx]

**Table 1- FDA approved indications for IV iron therapy ^(5)^**

| 1. Iron deficiency where oral iron administration is unsatisfactory or impossible  2. Iron deficiency anaemia in adult and paediatric Chronic Kidney Disease (CKD) patients receiving hemodialysis and receiving Erythropoietin Stimulating Agents  4. IDA in adult and paediatric patients with non-dialysis-dependent, hemodialysis dependent, and peritoneal dialysis-dependent CKD  5. IDA in adult patients with CKD  6. IDA in adult patients who have intolerance to oral iron or have had unsatisfactory response to oral iron or adult patients with non-dialysis-dependent CKD |
| --- |

* intestinal malabsorption can be detected by observing an increase in serum iron of less than 100 µg % over baseline in a fasting patient 1 or 2 hours after taking 60 mg iron as ferrous sulphate; CKD- Chronic Kidney Disease, ESA- Erythropoietic Stimulating Agent

**Table 2: Different parenteral iron formulations and their salient features**

|  | **Iron Dextran**  **(LMW)** | **Ferric gluconate** | **Iron Sucrose** | **Ferromoxytol** | **Ferric carboxy-maltose** | **Iron isomaltoside** | **Iron polymaltose** | **Ferric derisomaltose** |
| --- | --- | --- | --- | --- | --- | --- | --- | --- |
| **Molecular & Carbohydrate formulation** | Dextran | Gluconate | Sucrose | Semisynthetic Polyglucose sorbitol car-boxymethylether | Daiichi Sankyo Carboxymaltose | Isomaltoside (linear oligosaccharide) | Polymaltose | Derisomaltose |
| **Complex type** | Type I  Robust & strong | Type III  Labile weak | Type II  Semi-robust and moderately strong | Type I  Robust & strong | Type I  Robust & strong | Type I  Robust & strong | Type I  Robust & strong | Type I  Robust & strong |
| **Molecular weight (kD)** | LMW Dextran-165 | 289-440 | 30-60 | 750 | 150 | 150 | 50-80 | 34-60 |
| **Initial volume of distribution (L)** | 3.5 | 6 | 3.4 | 3.16 | 3.5 | 3.4 | - | - |
| **Plasma half-life (h)** | 20-24 | 1 | 5-6 | 15 | 7-12 | 20 | 22 | 27 |
| **Iron content (mg/mL)** | 50 | 12.5 | 20 | 30 | 50 | 100 | 50 | 100 |
| **Maximal single dose (mg)** | 2 mL undiluted iron dextran or 20 mg/kg (FDA) | 125-250 mg, as bolus or short infusion | 200-300, Maximum weekly dose 600 mg | 510 mg | 1000 mg Europe, 750 mg US | 20 mg/kg (max 2000 mg in one infusion) | Upto 1000 mg | 20 mg/kg (1000 mg in > 66 kg ) |
| **Premedication** | No | No | No | No | No | No | No | No |
| **Specific**  **advantage points** | Rapid and high-dose correction | Less risk of anaphylaxis (0.002%) | -Only approved for use in patients with CKD undergoing haemodialysis | - Rapid single dose infusion | -Controlled delivery of iron  -Single dose administration in short time  - Cost effective  -Very low immunogenic potential  - Safe in pregnancy | - Rapid one dose infusion  - Low immunogenic potential  -Less disruption of life, reduced physician and nurse time, Reduced treatment period, improved cost-effectiveness, etc.  -Higher ferritin level, delays the recurrence of IDA | - Single dose administration (up to 1000 mg)  - Inexpensive | -Short infusion time  -Only FDA approved IV therapy for single dose administration |
| **Specific**  **disadvantage points** | High risk of anaphylaxis,  Multiple hospital visit | Can cause hypotension (2%) | High risk of anaphylaxis,  Multiple hospital visit | High incidence of hypersensitivity  - High rate of tissue iron deposition | High risk of hypophosphatemia  - Transient interference with diagnostic ability of MRI  -Expensive  - May require repeated dosing | Can cause hypophosphatemia but usually incidence is low | Long infusion time | Expensive |
